# A mentor training workshop focused on fostering diversity engenders lasting impact on mentoring techniques: Results of a long-term evaluation

**DOI:** 10.1017/cts.2021.24

**Published:** 2021-03-16

**Authors:** Mallory O. Johnson, Jonathan D. Fuchs, Lauren Sterling, John A. Sauceda, Michael S. Saag, Alicia Fernandez, Clyde H. Evans, Monica Gandhi

**Affiliations:** 1 Department of Medicine, University of California, San Francisco, San Francisco, USA; 2 San Francisco Department of Public Health, University of California, San Francisco, San Francisco, USA; 3 Department of Medicine, University of Alabama, Birmingham, USA; 4 CE Consulting, Needham, USA

**Keywords:** Mentoring, diversity, HIV/AIDS

## Abstract

**Introduction:**

Trainees and investigators from underrepresented minority (URM) backgrounds face unique challenges to establishing successful careers in clinical and translational research. Structured training for mentors is an important mechanism to increase the diversity of the research workforce. This article presents data from an evaluation of the University of California, San Francisco (UCSF) Center for AIDS Research (CFAR) Mentoring the Mentors program aimed at improving mentors’ competency in working with diverse mentees in HIV research.

**Methods:**

Mentors from around the USA who had in one of seven separate 2-day training workshops conducted from 2013 to 2020 were invited to participate in an online evaluation survey of their experiences with the training and their subsequent mentoring activities.

**Results:**

There was a high response rate (80%) among the 226 mentors invited to complete the survey. The 180 respondents were diverse in demographics, professional disciplines, and geographic distribution. Quantitative and qualitative data indicate a lasting positive impact of the training, with sustained improvements documented on a validated measure of self-appraised mentoring competency. Respondents also endorsed high interest in future, follow-up training with continued focus on topics related to mentoring in the context of diversity.

**Conclusion:**

The evaluation of the UCSF CFAR Mentoring the Mentors program showed lasting impact in improving mentoring practices, coupled with high interest in continued in-depth training in areas focused on diversity, equity, and inclusion.

## Introduction

Researchers who are underrepresented minorities (URMs) in health-related sciences often face unique barriers in the development of independent research careers, including the absence or inadequacy of mentoring and research collaborations [1–8]. Historically, disproportionately fewer URM scientists than White scientists have been funded by the National Institutes of Health (NIH), Centers for Disease Control and Prevention (CDC), or other federal agencies, with NIH data indicating that Black and Latino scientists are significantly less likely to receive R01 funding than their White counterparts, despite equivalent training and publication records [9,10]. More recent data underscore this inequity, with heightened concern for women of color researchers [11]. Results of recent analyses showed that the topics disproportionately studied by Black researchers (associated with key words such as socioeconomic, health care, disparity, lifestyle, psychosocial, and risk) were less likely to be discussed at NIH study section than topics more likely studied by White researchers (e.g., key words that use more “biomedical” terms) [12].

The heightened challenges faced by trainees and academicians from underrepresented backgrounds are particularly pronounced in HIV research, as documented by an inadequate and fragile pipeline of diverse investigators focused on HIV priority areas [13].Given that the HIV epidemic in the USA is concentrated disproportionately among minority populations [14], this lack of representation among HIV researchers is particularly concerning. An effective response to ending the HIV epidemic must target prevention of both transmission and acquisition of HIV, as well as optimal implementation of evidence-based interventions to treat HIV and achieve the goals of therapy [15]. Often, however, the communities most impacted by HIV or HIV risk are underserved, under-resourced, and the least likely to have access to biomedical innovations, while being the most likely to experience competing social determinants that hinder use of or access to these innovations [16]. A well-trained and diverse scientific research workforce will help overcome some of these health disparities [17]. Reports underscore that researchers from racial and ethnic groups that are URMs in health-related sciences, and those non-URM scientists who are deeply familiar with marginalized communities, are particularly well situated to carry out this important work [18–27].

So, how do we develop a well-trained and diverse scientific research workforce? One way is through training mentors to more effectively mentor. The importance of effective and dedicated mentoring of early-stage investigators (ESIs) in academic research has been well documented, with growing evidence of the benefits of mentoring on productivity, job satisfaction, and quality of life [28,29]. However, most experienced investigators who are positioned to provide mentoring have not received formal training in mentoring techniques. Rather, they are expected to mentor without structure or training and often develop *ad hoc* methods and approaches that vary in consistency, intensity, and effectiveness [30]. With increased recognition of the importance of mentoring in academic research, some formal efforts are underway to develop, execute, and then evaluate mentor training curricula with the goal of improving relevant outcomes among mentors and mentees, as well as monitor improvements in mentoring competence [31,32].

The literature on mentoring offers some effective strategies and perspectives in designing effective mentoring training programs, including aligning mentor and mentee expectations and specifying roles early in the mentoring relationship [32–34]. Of particular importance is the need for consistent, tailored mentoring that takes into account the challenges faced by ESIs who are working to build their research careers. In recent years, the mounting barriers of unprecedented student loans, decreased availability of tenure-track positions, and declining funding for research have highlighted this need. For mentees from URM groups, this need is intensified.

Since 2012, the University of California, San Francisco (UCSF) Mentoring the Mentors program has conducted annual 2-day, in-person intensive mentoring training workshops to systematically build mentoring competency with an emphasis on training in techniques to mentor URM and otherwise diverse mentees in HIV research. The workshops are sponsored by the UCSF Center for AIDS Research (CFAR) with co-sponsorship from the CFAR Network of Integrated Clinical Systems (CNICS). The Mentoring the Mentors program focuses on HIV investigators based in the USA, as the program includes a focus on NIH and other funding opportunities available only to US investigators. The program curriculum, spanning two full days and led by faculty from a range of clinical and translational research disciplines and expert consultants, includes a combination of interactive, didactic, and case-based sessions that address pertinent issues in mentoring. These include an overview of principles and evidence for best mentoring practices, time and priority management, communication skills, how to mentor grant and paper writing, the role of individual differences (e.g., personality and leadership styles) on mentoring, and resources for mentoring more effectively. A prominent focus throughout the training is content and discussion related to mentoring across differences, with activities addressing implicit bias, microaggressions, and strategies to address barriers that uniquely affect research funding for URM ESIs. The operationalization of diversity largely followed the guidance from the NIH, which provides categories of individuals from racial and ethnic groups not well represented in science, persons with disabilities, and those from disadvantaged backgrounds. We extended this definition to include diversity along other dimensions, including gender identity, sexual identity, and religious or cultural views. Toward the end of each workshop, we implement a Mentor Consultation Clinic, in which the participants break into groups of 5–6 and provide input on one member’s current mentoring challenge. In this exercise, participants are instructed to apply the training content (e.g., active listening and awareness of bias) to a specific mentoring situation before offering advice and recommendations. See Table 1 for a sample agenda for the 2-day workshop.

**Table 1.** Mentoring the mentors workshop – sample agenda

Day 1
Definitions of Mentoring, Formal and Informal
Diversity, Mentoring, and Unconscious Bias
Introduction to Center for AIDS Research (CFAR) Network of Integrated Clinical Systems (CNICS) and opportunities for mentoring
Mentoring fundamentals – From Concept to Submission: Mentoring Scientific Writing
Introduction to resources and tools of mentoring as developed by the University of California, San Francisco’s Clinical and Translational Science Institute Mentoring Program
Mentoring fundamentals – From Concept to Submission: Mentoring Grant Writing
Mentoring fundamentals – Emotional intelligence
Personal Stories of Diversity
Day 2
Mentoring fundamentals – Self-Awareness in the Mentoring Relationship and Leadership Training
Mentoring fundamentals – Time management
Exercise on leadership styles
Mentoring fundamentals – Giving and receiving feedback
Mentoring Consultation Clinic

To date, we have trained over 226 HIV researcher mentors from across the USA, and the program and short-term evaluation results have previously been described [35–37]. Those who were included in these trainings were HIV researchers who were either already providing mentoring or who were far along enough in their careers to be positioned to take on substantive mentoring activities (e.g., not in a postdoctoral fellowship nor early assistant professor level). Before each workshop, we used multiple listservs and networks to solicit applications from US-based mentors, and the senior faculty reviewed applications for eligibility and fit for the program. Generally, we accepted all eligible applicants but rejected those who were too junior, who were not US-based, or who indicated not being in an academic research position that included mentoring. There were no registration costs for participation, and the program provided breakfast and lunch for each day as well as a group dinner after the first night. However, registrants were generally required to cover their own travel expenses with the exception of a limited number travel scholarships for a subset of applicants who indicated need; URM mentors were prioritized for these funds. After each workshop, we conducted confidential evaluations, the results of which guided the choice of topics and speakers for subsequent workshops. We also regularly updated the literature on diversity, equity, and inclusion that is covered in the training.

The purpose of this paper is to report longer-term mentoring outcomes from training program participants by assessing their perspectives once they have returned to their professional roles at their home institutions. In essence, we sought to answer the question of how successful was the program in enhancing participants’ ability to mentor diverse investigators in HIV research.

## Methods

### Participants and Procedures

To be eligible for this evaluation, individuals must have participated in one of seven in-person 2-day workshops between October 2012 and January 2020. From November to December 2020, a total of 226 unique participants who had attended at least one workshop were invited by email to complete a web-based survey via Qualtrics (Provo, UT). Following initial invitations, reminders were sent weekly to those who had not responded and those who had started, but not completed, the survey. To encourage participation, mentors who completed the survey received a US$10 gift code for an online retailer and were entered into a raffle for an additional one of five randomly awarded US$100 gift codes.

### Survey Content

The primary objective of the survey was to solicit data on the long-term impact of the program. The survey included questions about demographics, professional disciplines, current positions, and allocation of professional effort across domains (e.g., research, clinical, teaching, mentoring, and administrative). We then assessed perceived impact of the training on the participants’ mentoring using quantitative items (e.g., “As a result of participating in the Mentoring the Mentors program, I am more competent in addressing diversity, equity, and inclusion in my mentoring”) using 5-point Strongly Agree to Strongly Disagree Likert scale. These items were supplemented with an open-ended item that asked them to explain how the training impacted their mentoring using text entry responses.

As with prior reports of short-term impacts of the individual years’ workshops [35–37], the evaluation included the validated Mentoring Competency Assessment (MCA) [31].The MCA is a 26-item skills inventory that solicits self-appraisals of confidence in mentoring in six domains: 1) maintaining effective communication; 2) aligning expectations; 3) assessing understanding; 4) addressing diversity; 5) fostering independence; and 6) promoting professional development (Cronbach alphas = 0.62–0.91). Pre-workshop and immediate post-workshop MCA scores were previously collected for individual training workshops, and data from all years were merged for the current analyses. T-tests were computed to compare means of attendees’ self-ratings of mentoring skills across all six domains of the MCA pre- and immediate post-workshop (which was collected ahead of time by surveys) and means of the long-term post-workshop scores.

Finally, we queried interest in a potential follow-up “Mentoring 201” advanced workshop and solicited interest in specific topics that were presented to respondents as well as open-text fields, so that respondents could share their ideas for follow-up trainings.

## Results

### Response Rates

Of 226 mentors invited, 180 (80%) completed the survey. Of those who did not complete the survey, two (<1%) responded that they were now retired or were no longer in a mentoring role; 32 (14%) never responded to multiple invitations; 3 (1%) opened but did not complete the survey; 9 (4%) opted out of completing the survey (an option offered through the Qualtrics survey platform that indicates they received the invitation but chose not to participate); and one (<1%) had a non-functioning email address, and we were unable to locate more current contact information.

### Respondent Characteristics

**Fig. 1.**
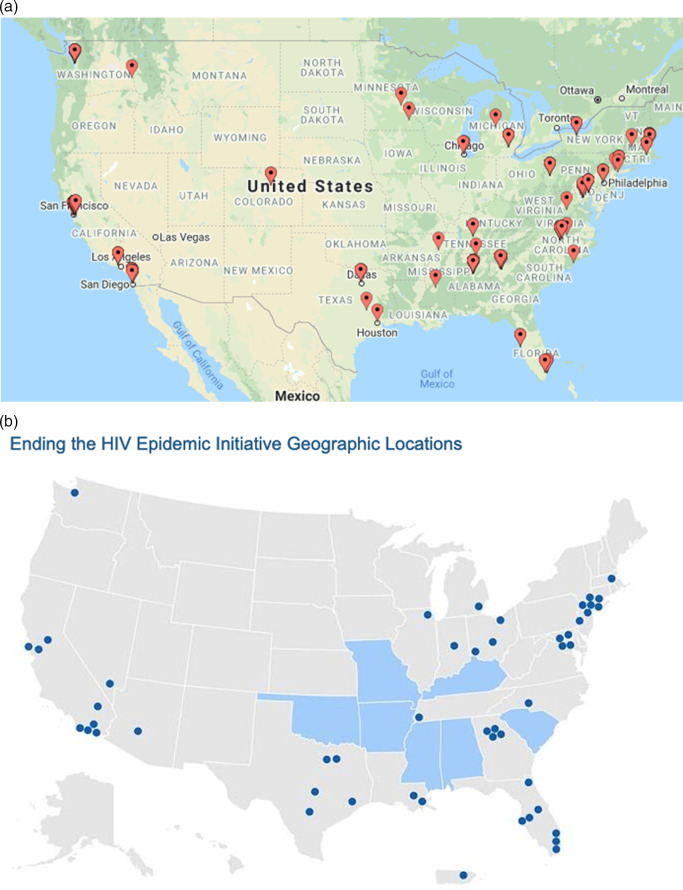
(a) Location of respondents’ primary institutions. (b) US Ending the HIV Epidemic priority states and counties [38].

Respondents’ primary work locations (based on zip code of their primary work institution) demonstrated geographic diversity throughout the US (see Fig. 1a) that corresponds to geographic priority areas in the US Ending the HIV Epidemic initiative (Fig. 1b) [38]. Table 2 presents the characteristics of the 180 mentors who completed the survey. Almost two-thirds were female, 12% Black/African American, 9% Asian, and just under 8% reported Latinx ethnicity. One-fifth reported being the first in their immediate families to attend college. Forty percent reported their primary discipline as medicine, followed by public health (26%), social/behavioral science (20%), and fewer than 10% each as basic science and nursing. At the time of this survey, most were at full (39%), associate (39%), and assistant (17%) professor ranks.

**Table 2.** Participant characteristics (N = 180)

Participant Characteristic		N (%)
**Gender identity**		
Female		115 (63.2)
Male		63 (34.6)
Gender minority		4 (2.2)
**Race** *		
Black or African American		23 (12.4)
Asian		17 (9.1)
Native Hawaiian or Other Pacific Islander		0
American Indian/Alaska Native		2 (1.1)
White		131 (70.4)
Another race or ethnicity		10 (5.6)
**Latino/a/x or Hispanic ethnicity**		14 (7.8)
**First in family to attend college**		36 (20.1)
**Primary discipline**		
Medicine		72 (40.0)
Social or Behavioral Science		36 (20.0)
Public Health		47 (26.1)
Basic Science		12 (6.7)
Nursing		9 (5.0)
Other		4 (2.2)
**Current academic rank**		
Assistant professor		31 (17.0)
Associate professor		71 (39.0)
Professor		71 (39.0)
Emerita/Emeritus		3 (1.7)
Other		6 (3.3)
**In mentoring/training leadership role**		115 (65.3)

	Mean (SD)	Range
**Proportion of time typically spent**		
Mentoring	13.6% (11.3)	0–80%
Teaching	9.9% (11.2)	0–70%
Research	55.5% (21.4)	0–100%
Clinical	7.2% (11.4)	0–70%
Administrative	12.7% (13.8)	0–70%
**Number of primary mentees**	4.6 (4.2)	0–36
**Number of secondary mentees**	5.0 (3.8)	0–25
**Year completed mentor training**		**N (%)**
	2012	8 (4.4)
	2013	27 (15.0)
	2015	29 (16.1)
	2017	30 (16.7)
	2018	40 (22.2)
	2019	30 (16.7)
	2020	23 (12.8)
	More than 1 year	5 (2.8)

* For race, Ns do not add up to total, as individuals could select more than one option.

### Mentoring Activities

Approximately two-thirds reported currently being in a leadership role related to mentoring or training. These included directing training programs, leadership of mentoring or developmental cores for research centers, and positions in academic affairs. Twenty-two percent reported having changed institutions or positions since participating in the training. Of those, 71% reported that their new position offered greater opportunities for mentoring. Since participating in the training, one-third reported having received an award related to their mentoring and an additional 15% reported that they were aware of being nominated for such an award. Mean number of current primary and secondary mentees were 4.6 and 5.0, respectively.

Mentors reported working with diverse mentees. Specifically, the following percentage of mentors reported working with mentees from the following groups: 98% with mentees who are female; 96% with mentees from racial or ethnic minority backgrounds; 79% with mentees from socioeconomically disadvantaged backgrounds; 46% with mentees who identify as gender minorities; 83% with mentees who identify as sexual minorities; 27% with mentees with disabilities; and 74% with mentees who are the first in their immediate families to attend college.

### Impact of Training

**Fig. 2.**
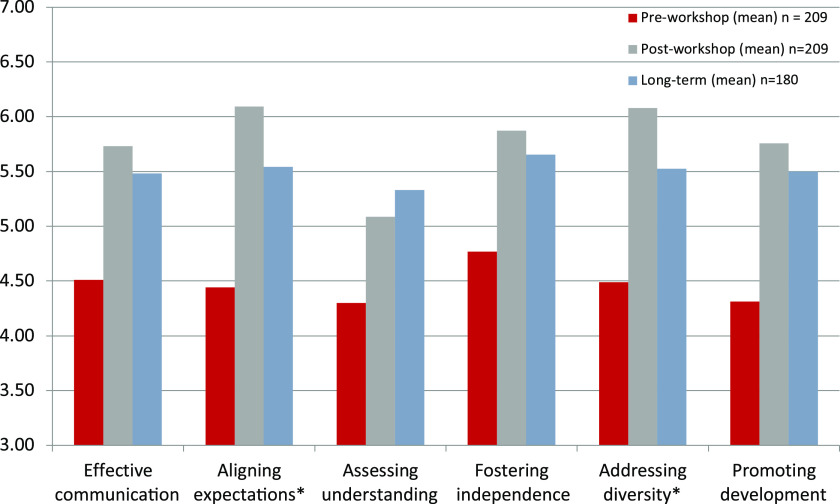
Changes in Mentoring Competency Assessment scores. *Note:* Mentoring Competency Assessment scale is from 1 to 7: all pre-immediate post- and pre-long-term diffences *P* < 0.001. *Immediate post-long-term differences *P* < 0.0001: all other immediate post-long-term differences not significant at *P* < 0.05.

Scores on all six subscales of the MCA showed increases from pre-workshop to immediate post-workshop levels, with sustained increases at long-term follow-up (Fig. 2). While long-term results revealed a modest reduction on most domains from the immediate post- to long-term post, only scores on the Aligning Expectations and Addressing Diversity subscales were significantly below immediate post-training levels (but importantly were still significantly higher than pre-training levels), consistent with a sustained improvement from baseline but somewhat diminished effect over time in these two domains. When asked about the impact of the mentoring workshop (Table 3), respondents indicated that the workshop had a lasting positive impact on their mentoring (92%), that they believe themselves to be a more effective mentor (91%), and to be more aware of the need for (82%) and more competent in (82%) addressing diversity, equity, and inclusion in their mentoring. More than 91% report having recommended the program to others, 58% had replicated or were planning to replicate training elements at their home institutions, 74% reported helping others with their mentoring skills, and 50% reported having kept in touch with others from their training workshop cohort.

**Table 3.** Reported impact of training

As a result of having participated in the Mentoring the Mentors workshop	Disagree or strongly disagree n (%)	Neutral n (%)	Strongly agree or agree n (%)
The workshop has had a lasting positive impact on my mentoring	2 (1.11)	13 (7.22)	165 (91.67)
I believe I am a more effective mentor	2 (1.11)	14 (7.78)	164 (91.11)
I am more aware of the need to address diversity, equity, and inclusion in my mentoring	6 (3.33)	26 (14.44)	148 (82.22)
I am more competent in addressing diversity, equity, and inclusion in my mentoring	6 (3.33)	35 (19.44)	139 (77.22)
I have recommended the program to others	3 (1.67)	13 (7.22)	164 (91.11)
I have replicated or plan to replicate parts of the training at my home institution	26 (14.61)	48 (26.97)	104 (58.43)
I have helped others develop their mentoring skills	17 (9.44)	29 (16.11)	134 (74.44)
I have kept in touch with others whom I met/interacted with in the training	52 (28.89)	38 (30.00)	90 (50.00)

### Qualitative Comments

Narrative comments reinforce these findings. Some referred to the unique opportunity to learn mentoring skills, noted as a gap in their prior training.

*“Being a good mentor is not something we are taught in medical school. It was a very powerful experience to have the chance to reflect on my mentorship style and learn some practical tips for improving my skills.”* Woman, White, Medicine, Associate Professor.

and

*“It was a tremendous opportunity to focus on a critical area of professional development that is a rare focus. Academics are expected to ‘know’ how to effectively mentor and support mentees in their own career development but rarely in our own education and career development do we get support, knowledge and training on ‘how’ to most effectively mentor others. This is why this program is invaluable.”* Woman, Middle Eastern/North African, Social/Behavioral Science, Professor.

and

*“I consider mentorship to be one of the most important aspects of my job. But in early-career I felt thrown into these relationships with minimal instruction, oversight, and constructive feedback. The Mentoring the Mentors program provided a sense of community of a group of professionals who are striving to do mentorship better and the encouragement of leaders and experts who desire to foster equity and excellence in mentorship.”* Woman, White, Public Health, Associate Professor.

Some identified the value of specific tools covered in the training.

*“I learned that there is a science in mentoring and that as a mentor I need to be more systematic.”* Woman, Black/Latinx, Social/Behavioral Science, Professor.

and

*“The Mentoring the Mentors workshop helped me better understand my own communication style and how to effectively work with mentees who have different backgrounds from me. I also got some excellent concrete tools to use in my own mentoring, such as mentoring agreements and worksheets to guide weekly meetings with mentees.”* Woman, White, Medicine, Associate Professor.

and

*“I appreciated the way the workshop approached the complex role of mentoring into a smaller subset of skills and tools that seemed easier to engage with. The instructors were all extremely engaged and engaging, and shared specific examples of mentoring challenges and strategies that were immediately applicable to my own work with mentees.”* Man, White, Public Health, Professor.

Some referred to the value of the safe environment cultivated in the workshops

*“Above all, the course is conducted in a supportive, creative, and fun environment that helps attendees feel ‘safe’ in expressing their opinions in order to learn from each other.”* Man, White, Medicine, Associate Professor.

and

*“The Mentoring the Mentors facilitators created a supportive, dynamic, thought-provoking environment in which to explore my own pre-conceptions and think about different learning styles. It was energizing to share ideas with other people who want to be better at supporting the next wave of researchers and really recognizing and developing their talents and strengths.”* Woman, White, Social/Behavioral Science, Research Scientist.

Others noted the importance of the training focus on diversity, equity, and inclusion

*“This workshop helped me to think about the challenges that diverse mentees face and to come up with strategies to help them succeed.”* Man, White, Public Health, Professor.

and

*“Mentoring diverse trainees requires specific skills, self-awareness, and dedication. The Mentoring the Mentors program was a critical step in my journey towards becoming a better and more effective mentor for trainees of diverse backgrounds.”* Woman, White, Social/Behavioral Science, Professor, first in family to attend college.

and

*“The UCSF Mentoring the Mentors program expands beyond stressing the importance of increasing diversity in mentors and mentees to effectively teach mentors how to continuously be mindful of and address biases that are inherent in the mentor-mentee relationship. This is a skill that is critical in fostering a productive and successful mentor-mentee relationship.”* Woman, Black, Epidemiology, Associate Professor, first in family to attend college.

### Interest in a Future Advanced Training

When asked whether respondents would be interested in a follow-up advanced training (Mentoring 201), a majority reported being definitely (68%) or probably (26%) interested. When asked to rate interest in specific topics, those that received the highest ratings were 1) mentoring during times of stress and uncertainty; 2) best practices for distance mentoring; and 3) racism/sexism/xenophobia/discrimination as they relate to mentoring (82%, 79%, and 74%, respectively, endorsed being very interested). Other topics offered by survey respondents included a focus on mentor self-care (e.g., setting limits, preventing burnout), co-mentoring best practices, training mentees to be effective mentors, working with challenging or stuck mentees, mentoring mentees from different disciplines, mentoring mentees from low- and middle-income countries, and improving one’s institutional culture and commitment to mentoring.

## Discussion

Effective mentoring is crucial to the success of ESIs; training programs in mentoring are limited and enhancing mentoring skills via training may be particularly impactful for mentees from URM groups. We report on combined outcomes from 7 years of a designated mentoring training program with a specific focus on diversity. The results of this evaluation show sustained effects on mentoring competency among mentors who participated in the program. Of note is that the mentors are now working with a diverse range of mentees, including racial, ethnic, sexual, gender minorities; mentees with disabilities; and mentees who were the first in their immediate families to attend college. This is highly encouraging given the need to increase the diversity of the pipeline of investigators focused on research to end the HIV epidemic. It is also encouraging that the mentors trained through the Mentoring the Mentors program are generally based in geographic areas that harbor the highest prevalence and incidence of HIV in the USA (Fig. 1a and b).

Results suggest that the impact of the mentor training may extend beyond the immediate orbit of each participant’s direct mentoring pool, as a substantial proportion of workshop participants went on to hold leadership positions related to mentoring, to replicate what they learned in the training, or otherwise help others improve their mentoring effectiveness. In addition, we anticipate that improvements in participants’ mentoring practices will allow them to serve as role models to their mentees who will likely emulate their mentors as they develop their own mentoring practices. This formal and informal dissemination of the training offers promise for extending the reach of the program well beyond the pool of mentors who directly participated in the workshops.

The emphasis on mentoring across differences is central to the UCSF CFAR’s Mentoring the Mentors program. Addressing diversity is one of two areas, however, that showed a less sustained impact in mentoring practices over time. There are several speculative explanations for this finding. First, it may be that there has been heightened awareness of issues of diversity, equity, and inclusion as they relate to mentors in the decade since the program started. Second, the long-term survey occurred at the end of 2020, a year in which social, political, and public health issues brought multiple dimensions of disparities and discrimination into the public awareness. This salience may have resulted in respondents being more aware of their need to better support mentees from underrepresented backgrounds in their mentoring. Finally, it may be that our curriculum must continue to evolve to have greater impact on the capacity of mentors to address diversity in their mentoring practices. Indeed, we are refining our current program and developing an advanced mentor training program in which we expand our focus on diversity, including bringing in content related to intersectionality in the mentoring relationship as well as working with mentees during times of stress and uncertainty. In response to survey feedback, we are also developing a module on remote mentoring best practices, a topic of necessity during the COVID-19 pandemic but which has relevance beyond that point as more mentors take on mentees who are geographically distant.

There are limitations that should be considered when generalizing the results of this evaluation. The mentors surveyed in this study had self-selected to both previously participate in an intensive in-person mentor training workshop and also agreed to complete this survey when invited. Therefore, the current sample may represent a group that is particularly dedicated to mentoring ESIs in HIV research. We also note that there was disproportionate representation among female mentors (almost two-thirds) in our trainings and in this evaluation. It is unclear whether this suggests a pattern of higher interest among female mentors in seeking help in improving their mentoring skills over the level of interest among their male counterparts; this warrants future investigation. Likewise, although we had a high response rate (80%), those who did not respond may have different perspectives or may have been particularly affected by the COVID-19 pandemic to the extent that they did not have the time or bandwidth to participate (e.g., some were likely pulled away for clinical work or research related to COVID-19). Similarly, the pool of mentors surveyed reflected investigators who, at least at the time of their participation in the Mentoring the Mentors program, were based at US institutions, and thus we have not captured the perspectives of investigators working primarily in low- and middle-income countries. While the MCA is a validated instrument, all other items were developed for this evaluation only and therefore do not have prior validation. The online survey format is well suited for quantitative data collection but may not be as effective in soliciting qualitative responses; the relatively brief responses provided in response to the open-ended inquiries may not have fully captured the rich and nuanced underlying themes that might have emerged through in-depth interviews with structured probing of relevant content. Our evaluation did not include the perspectives of mentees who work with the mentors trained in our program; including their perspectives and objective metrics of mentee successes would be valuable in future work to document the impact of the training. Finally, the data represent a narrow slice in time during an active phase of the unprecedented COVID-19 crisis, which may have altered the perceptions of respondents in a way that may not reflect their longer-term perspectives. In spite of these constraints, we contend that the findings presented in this study offer a unique and valuable contribution to the mentoring literature and illuminate strategies and opportunities for future mentor training programs.

In conclusion, our designated mentoring program for HIV researchers had long-term effects on mentoring competency. The data from this study support the impact and ongoing need for specialized training in tools and techniques of effective mentoring among researchers. Our results also support an increased focus on dynamics related to diversity, equity, and inclusion in mentoring trainings to help cultivate and maintain a diverse pipeline of future researchers committed to clinical and translational research. Although this paper focuses on the perspectives of mentors working primarily in HIV research, our findings are not confined to that context. Rather, the results are generalizable to the challenges and opportunities for mentoring across a wider range of academic research settings. Given the interest in subsequent and more advanced workshops, it is also apparent that growth in mentoring skills is an ongoing process, and that systematic support and evidence-based training for mentors should be provided throughout mentors’ careers.
